# The management of retrorectal tumours: tertiary centre retrospective study

**DOI:** 10.1093/bjsopen/zrac044

**Published:** 2022-04-20

**Authors:** Joshua R. Burke, Kunal Shetty, Owen Thomas, Mikolaj Kowal, Aaron Quyn, Peter Sagar

**Affiliations:** The John Golligher Colorectal Surgery Unit, St. James’s University Hospital, Leeds Teaching Hospital Trust, Beckett Street, Leeds, UK; Leeds Institute of Biomedical & Clinical Sciences, Clinical Sciences Building, St James’s University Hospital, Leeds, UK; The John Golligher Colorectal Surgery Unit, St. James’s University Hospital, Leeds Teaching Hospital Trust, Beckett Street, Leeds, UK; Leeds Institute of Biomedical & Clinical Sciences, Clinical Sciences Building, St James’s University Hospital, Leeds, UK; The John Golligher Colorectal Surgery Unit, St. James’s University Hospital, Leeds Teaching Hospital Trust, Beckett Street, Leeds, UK; Leeds Institute of Biomedical & Clinical Sciences, Clinical Sciences Building, St James’s University Hospital, Leeds, UK; The John Golligher Colorectal Surgery Unit, St. James’s University Hospital, Leeds Teaching Hospital Trust, Beckett Street, Leeds, UK; Leeds Institute of Biomedical & Clinical Sciences, Clinical Sciences Building, St James’s University Hospital, Leeds, UK; The John Golligher Colorectal Surgery Unit, St. James’s University Hospital, Leeds Teaching Hospital Trust, Beckett Street, Leeds, UK; Leeds Institute of Biomedical & Clinical Sciences, Clinical Sciences Building, St James’s University Hospital, Leeds, UK; The John Golligher Colorectal Surgery Unit, St. James’s University Hospital, Leeds Teaching Hospital Trust, Beckett Street, Leeds, UK; Leeds Institute of Biomedical & Clinical Sciences, Clinical Sciences Building, St James’s University Hospital, Leeds, UK

## Abstract

**Aim:**

Tumours of the retrorectal space are uncommon, pathologically heterogeneous, and difficult to diagnose, with ongoing controversy over their surgical management. The aim of this study was to evaluate the surgical management of a consecutive series of patients who had undergone excision of primary retrorectal tumours (PRRTs) at a tertiary referral centre.

**Method:**

Patients were identified from a prospectively maintained database between 1 March 2001 and 1 August 2021. Electronic patient records were reviewed for demographics, preoperative imaging, operative details, histology, and follow-up. A chi-squared test was used to assess the statistical significance of findings.

**Results:**

A total of 144 patients were included in the study. Of these, 103 patients were female (71.5 per cent), 46 patients (31.9 per cent) presented incidentally, and 99 of the patients had tumours located below S3 (68.7 per cent). Overall, 76 patients underwent a transperineal approach (52.7 per cent) with the most common findings of a benign tailgut cyst occurring in 59 (40.9 per cent) of cases. Preoperative MRI predicted urovascular and pelvic sidewall involvement assessed intraoperatively with a sensitivity of 83.3 and 90 per cent and a specificity of 98.1 and 98 per cent respectively. Risk of malignancy in solid tumours was 31.4 *versus* 8.8 per cent in cystic tumours (relative risk 3.5, 95 per cent c.i. 1.6 to 7.6, *P* < 0.001). Major complications (Clavien–Dindo grade III and above) occurred in eight patients (5.6 per cent) and all-cause long-term mortality was 4.8 per cent (seven patients).

**Discussion:**

PRRTs can be safely excised with minimal complications in specialized centres by surgical teams with the relevant expertise. This study questions the conservative management of cystic tumours and given the risk of solid tumour malignancy, supports surgical management.

## Teaser

There remains ongoing controversy over the surgical management of retrorectal tumours. The manuscript demonstrates that these tumours can be safely excised with minimal complications and brings into question the conservative management of cystic tumours given the risk of solid tumour malignancy.

## Introduction

Tumours of the presacral or retrorectal space are uncommon, pathologically heterogeneous, difficult to diagnose^1^, and there is widespread controversy over their surgical management. The true incidence of this tumour type in unknown, but they are estimated to account for 1 in every 40 000–60 000 hospital admissions, with most patients being female with a median age of 45 years^2,3^. The retrorectal space contains multiple embryological remnants and diverse tissue types with pluripotent capability. They can be classified as benign, malignant, solid, and cystic, or categorized as congenital, inflammatory, neurogenic, osseous, and miscellaneous^1^.

Presentation and diagnosis of retrorectal tumours is often incidental with 26–50 per cent of patients being asymptomatic. Those that do present, do so with poorly localizing symptoms secondary to pelvic organ invasion or nerve compression^4^, the most common being non-specific chronic pain^5^.

Tumour diversity, anatomical complexity, and rarity of disease gives rise to a challenging surgical problem, managed by very few tertiary centres internationally and with reported substantial morbidity and risk of inappropriate management^6^. CT and MRI can be used for preoperative planning and is effective at differentiating between benign and malignant pathologies^5,7,8^.

Following diagnosis, patients undergo surgery to alleviate symptoms and prevent malignant transformation^2,9–11^; however, this may not be necessary in all cases with some evidence suggesting cystic lesions without suspicious radiological features can be followed by serial imaging without resection^12^. A recent systematic review by Baek *et al.* in 2016 included 341 studies involving 1708 patients, which supported complete surgical resection and where feasible through minimal access surgery^13^. The largest case series included was performed by Jao *et al.* in 1985 involving 120 patients^14^.

The aim of this study was to evaluate the surgical management of a consecutive series of patients who had undergone excision of primary retrorectal tumours (PRRTs) at a tertiary referral centre.

## Method

A prospectively maintained database identified patients who had undergone PRRT excision between 1 January 2001 and 1 August 2021 at St James Teaching Hospital (a tertiary referral centre in Leeds, UK). Electronic patient records were reviewed for demographic information, preoperative presentation, previous surgery, preoperative biopsy and imaging, anatomical location, size, surgical approach, histology, surgical complications, recurrence, and mortality. Postoperative complications were grouped according to the Clavien–Dindo classification (CDC)^15^. Patients with Ewing’s sarcoma, lymphoma, metastatic carcinoma, osteogenic sarcoma, recurrent rectal or anal cancer, or with unknown histology were excluded from the study. Individuals with recurrent retrorectal tumours, or those who had already undergone surgery, were also excluded from the analysis. All data were anonymized and collated with Microsoft^®^ Excel (Microsoft, San Diego, USA). The study met the local research committee criteria for audit of practice.

### Tumour position and imaging

Tumour position was described for each patient by way of the imaging modalities outlined below. The position was correlated with the surgical approach utilized for each patient.

**Fig. 1:**
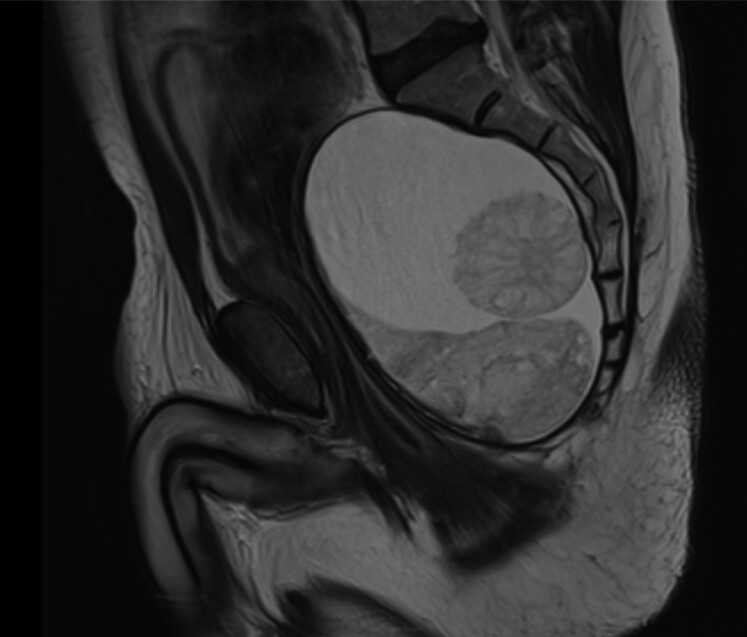
MRI of the pelvis and rectum demonstrates a large well defined cystic lesion filling the entire lower pelvis with intermediate-to-high signal, likely solid components antero-inferiorly but no invasive margin. Histopathology showed benign tailgut cyst with previous haemorrhage.

**Fig. 2:**
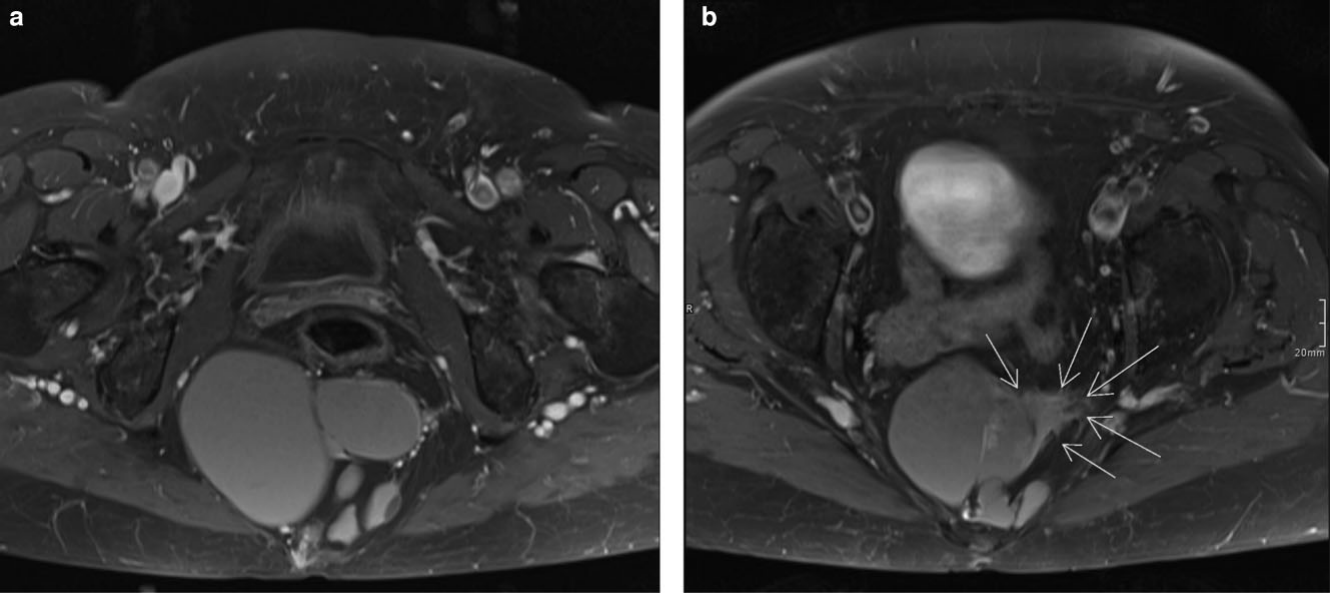
**Transformation of a tailgut cyst from a cystic appearance (a)** to a mixed heterogenous component marked with arrows **(b)** on surveillance MRI axial images.

All patients had MRI (*Figs 1* and *2*) unless contraindicated, in which case CT was performed. Where malignant lesions were suspected, a CT of the chest and abdomen was also performed. Preoperative imaging was routinely reviewed for the following features^16^:

- Proximity/involvement of rectum/mesorectal fascia
- Relationship to sacral bodies and nerve roots
- Proximity to iliac vessels
- Involvement of pelvis viscera and/or pelvic sidewall structures
- Relationship to greater sciatic notch

MRI imaging was used to predict urovascular and pelvic sidewall involvement. To analyse the accuracy of this, intraoperative findings of the above were correlated with scan results. If urovascular or pelvic sidewall involvement was noted on imaging and confirmed subjectively by the operating surgeon intraoperatively, this was considered an accurate prediction. The accuracy was analysed through calculations of sensitivity, specificity, positive predictive value (PPV), and negative predictive value (NPV).

### Management

The tertiary referral centre does not routinely perform preoperative biopsy but an examination under anaesthetic is performed if required, to confirm preoperative imaging, and inform the surgical management plan. All patients received full preoperative bowel preparation. Due to the heterogeneous nature of retrorectal tumours, surgical procedures vary considerably. The group has previously published a management algorithm on primary^17^ and recurrent retrorectal tumours^16^, and is described in brief here:

- The surgical management involves three approaches: perineal, abdominal, and combined.
- The surgical approach is determined by tumour position, involvement of the sacrum, adjacent viscera, or pelvic sidewall, benign or malignant tumour appearance, and its size.
- The principal factor in surgical decision-making is tumour position, with tumours below the mid-body of S3 being predominantly amenable to a perineal approach.
- The approach is supported by clinical examination: if the equator of the tumour can be palpated on digital rectal examination, a perineal approach can be considered.
- All tumours extending above S3 require an abdominal or combined approach.

### Statistical analysis

All descriptive statistics were conducted with SPSS^®^ version 28 (IBM, Armonk, New York, USA). Sensitivity, specificity, NPV, and PPV were calculated for preoperative imaging’s ability to successfully predict urovascular and pelvic sidewall involvement of the tumour respectively. A chi-squared test was used to assess the statistical significance of the risk of malignancy between cystic and solid tumours, the risk of recurrence between R0 and R1 resection, and the risk of death between symptomatic and incidental presentation.

## Results

A total of 182 cases were initially identified from the prospectively maintained database between 1 March 2001 and 1 August 2021. Four patients had histological results that excluded them from the study, 23 had recurrent retrorectal tumours, 10 had missing data, and 1 patient did not attend their operation and was lost to follow-up. The remaining 144 patients met the study criteria and were included for analysis. Of these, 103 patients were female (71.5 per cent). The median (interquartile range; i.q.r.) age at operation was 49 (37– 61) years.

### Presentation

46 patients (31.9 per cent) presented incidentally. Of these patients, 19 (41.3 per cent) were identified through either CT or MRI imaging, and 9 (19.6 per cent) through ultrasound and diagnostic laparoscopy for unrelated conditions. The remainder of incidental presentations were referred following other diagnostic findings. Overall, 98 patients (68.1 per cent) were symptomatic at presentation. Of these symptomatic patients, 39 (39.7 per cent) presented with pelvic pain or discomfort, 17 with a mass (17.3 per cent), 8 with change in bowel habit (8.1 per cent), and 9 with obstructive defaecation (9.1 per cent). Other less frequent presentations included back pain, neurological symptoms, perianal abscess, and weight loss.

### Tumour position

A total of 99 tumours were below S3 (68.7 per cent), 18 were at S3 or above (12.5 per cent), and 27 were above and below S3 (18.75 per cent). The median (i.q.r.) size of maximal dimension was 6.5 (1–12) cm and ranged 1–23 cm, measured during macroscopic histological assessment.

### Surgical approach

**Fig. 3:**
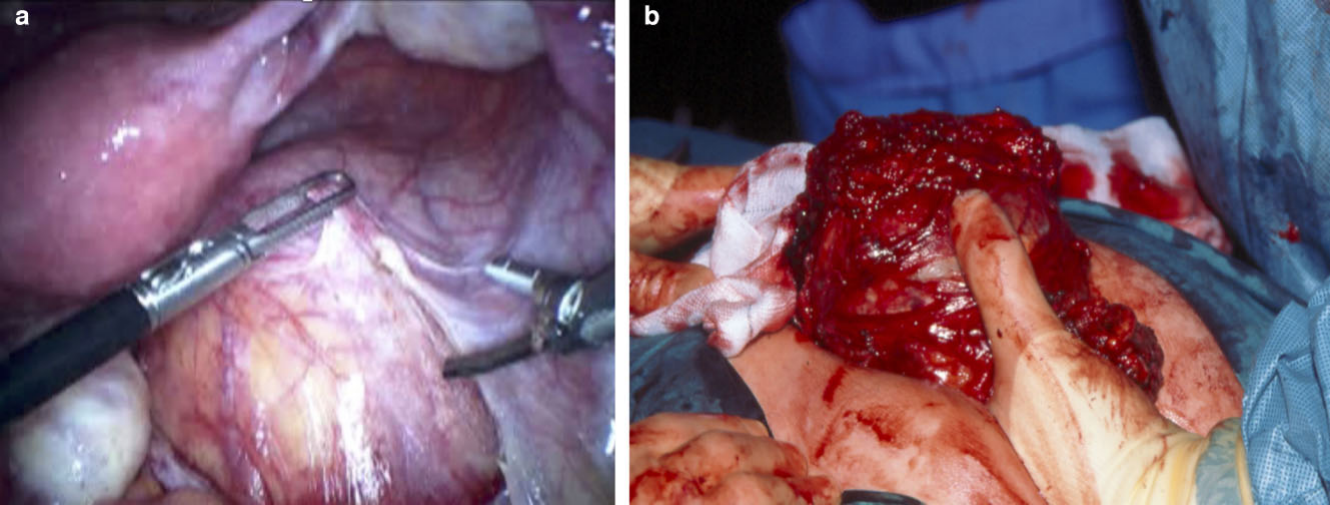
**a** Displays an intra-abdominal image of initial laparoscopic dissection around a retrorectal Schwannoma. **b** Depicts excision of chordoma that required a distal sacrectomy via a perineal approach with the patient in a prone jack-knife position.

A total of 76 (52.7 per cent) patients underwent a transperineal approach (*Fig. 3*), 59 underwent an abdominal approach (40.9 per cent), and 9 underwent a combined approach (6.2 per cent). Of the patients who had an abdominal approach, 25 were laparoscopic (42.3 per cent). Two patients required conversion to open (8 per cent).

The factors necessitating an abdominal or combined approach were location of tumour in 46 patients (67.4 per cent), organ involvement in 10 patients (14.7 per cent), history of previous surgery in 6 patients (8.8 per cent), pelvic side wall involvement in 3 patients (4.4 per cent), other pathology present requiring excision in 2 patients (2.9 per cent), and size of tumour in 1 patient (1.5 per cent).

### MRI preoperative accuracy:

MR imaging demonstrated a sensitivity of 83.3 per cent and a specificity of 98.1 per cent in predicting whether retrorectal tumours were likely to have any urovascular involvement. In this population, the PPV was 71.4 per cent, and the NPV was 99.4 per cent. Six patients were found to have actual urovascular involvement.

When predicting whether the tumour had any pelvic sidewall involvement for 10 patients who had actual involvement, MRI was found to have a sensitivity of 90 per cent and a specificity of 98 per cent. In this population, the PPV was 75 per cent and the NPV was 99.3 per cent.

### Surgical sequalae

Operative blood loss was minimal in 105 patients (72.9 per cent), less than or equal to 250 ml in 21 patients (14.5 per cent), less than 1 litre in 12 patients (8.3 per cent), and more than 1 litre in 6 patients (4.1 per cent). All cases with blood loss greater than 250 ml occurred in patients who had an abdominal or combined approach.

Organ resection was required in 22 (15.2 per cent) patients. If excised, the rectum was the most common organ (14–9.7 per cent), followed by the coccyx (6–4.1 per cent), and the sacrum (6–4.1 per cent). Four patients had a combination of these organs removed (2.7 per cent). Seventeen patients required a stoma at operation (11.8 per cent). At the time of analysis, 4 patients have had their stoma reversed (2.7 per cent), 12 have not been reversed (8.3 per cent), 2 had a stoma before the operation (1.3 per cent), and 1 had a failed reversal (0.7 per cent).

The median (i.q.r.) duration of hospital of stay was 5 (3–8) days, with a range of 1–76 days. The duration of hospital stay was unavailable for three patients. Five patients required readmission (3.4 per cent), one for abdominal pain that was negative for a cause on CT, one for treatment of cyst recurrence, one for a lower respiratory tract infection, one for symptoms regarding an inserted pelvic drain, and one for pain control.

No and minor complications (defined as CDC grade I and II) were observed in 111 (77.1 per cent) and 22 (15.2 per cent) patients respectively (*Table 1*). Major complications (CDC grade III and above) occurred in 11 patients (7.6 per cent). Out of the major complications, seven patients required radiological interventions (4.8 per cent), two patients required a relaparotomy for removal of packing (1.3 per cent), one patient developed myocardial infarction (0.7 per cent), and one patient died because of acute renal failure (0.7 per cent).

**Table 1:** Early complications are tabulated by Clavien-Dindo classification (CDC)

Type	*n* (%)	Cause (*n*)
**None**	114 (77.1%)	
**CDC I**	11 (7.6%)	Prolonged stay/readmission due to pain (5), superficial wound dehiscence (3), neuropraxia (1), urinary retention (1) and erectile dysfunction (1).
**CDC II**	11 (7.6%)	Wound infection requiring antibiotics (6), osteomyelitis (1), chest infection (1), chronic perineal sinus (1) and blood transfusion (2)
**CDC III**	9 (6.2%)	Radiological drainage of pelvic/abdominal collection (6), nephrostomy (1), relook laparotomy for pelvic packing (2)
**CDC IV**	1 (0.7%)	Myocardial infarction (1)
**CDC V**	1 (0.7%)	Death (1)

### Histology

A total of 14 (9.7 per cent) patients underwent preoperative biopsy. When biopsy was undertaken there was discordance with the biopsy histology results and the final specimen histology after resection in four cases (28.6 per cent). Of these cases, one suggested fibrosis that was found to be a fibrous attachment of endometrial carcinoma, two cases were reported as healthy tissue that was found to be schwannomas, and one biopsy result suggested repair tissue that was found to be a leiomyoma on final histology.

The most common indication for resection was a tailgut cyst; the majority were benign in 59 (40.9 per cent) cases. Malignant transformation within the tailgut cyst was observed in two cases detecting adenocarcinoma, one with squamous cell carcinoma (SCC), and another case showing a transitional cell carcinoma. Nineteen schwannomas were identified (13.1 per cent) and 17 epidermoid cysts (11.8 per cent), 1 being just adjacent to a neuroendocrine carcinoma, and another containing SCC *in situ*. Nine chordomas were identified (6.3 per cent), one of which was sarcomatoid.

After histological analysis (*Table 2*), 25 tumours were found to be malignant (17.3 per cent) and 119 were benign (82.7 per cent). A total of 90 tumours were cystic in nature (62.5 per cent) and 54 were solid (37.5 per cent). Of the cystic tumours, 91.1 per cent were benign (*n* = 82), whereas 68.5 per cent of solid tumours were benign (*n* = 37). Risk of malignancy in solid tumours was 31.4 *versus* 8.8 per cent in cystic tumours (relative risk 3.5, 95 per cent 1.6–7.6 *P* < 0.001).

**Table 2:** Distribution based on excised histopathological analysis

Type	Histology	*n* (%)
**Malignant**	Chordoma	8 (5.5%)
	Gastrointestinal stromal tumour	3 (2.1%)
	Adenocarcinoma in tailgut cyst	2 (1.3%)
	Adenocarcinoma in duplication cyst; endometrial carcinoma; epidermoid with squamous cell carcinoma; liposarcoma grade I; serous carcinoma high grade; leiomyosarcoma high grade; pleomorphic rhabdomyosarcoma; Malignant degeneration of tailgut cyst; neuroendocrine tumour adjacent to epidermoid cyst; sarcomatoid chordoma; squamous cell cancer; transitional cell cancer in tailgut cyst	1 (0.7%) each
	Total	25 (17.3%)
**Benign**		
	Tailgut cyst	59 (40.9%)
	Schwannoma	19 (13.1%)
	Epidermoid cyst	15 (10.4%)
	Cystic teratoma	4 (2.7%)
	Leiomyoma	4 (2.7%)
	Myelolipoma	3 (2.1%)
	Ganglioneuroma; lipoma; fibrosis/fibrotic tumour	2 (1.3%) each
	angiomyxoma; benign mucin-secreting tumour; chronic inflammatory cyst; cystic lymphangioma; foreign-body giant-cell reaction; lymph nodes foreign-body reaction; teratoma; mesothelial cyst; rectal duplication cyst	1 (0.7%) each
	Total	119 (82.6%)

### Recurrence and mortality

R0 resection was achieved in (92 per cent) of patients with malignant tumours. In both benign and malignant tumours an overall recurrence rate of 13.2 per cent (*n* = 19) was observed (median (i.q.r.) follow-up of 82 (47–126) months). Of those tumours that recurred, 6 (31.5 per cent) were malignant and 13 (68.5 per cent) were benign, 12 (63 per cent) were cystic and 7 (37 per cent) were solid. According to histological subtype, all epidermoid cysts with SCC (*n* = 1), cystic lymphangioma (*n* = 1), and pleomorphic rhabdomyosarcoma (*n* = 1), 50 per cent of lipomas (*n* = 1), 37 per cent of chordomas (*n* = 3), 25 per cent of cystic teratomas (*n* = 1), and 50 per cent of tailgut cyst with adenocarcinoma (*n* = 1), 12 per cent of benign tailgut cysts (*n* = 7), 11 per cent in schwannomas (*n* = 2), and 6.7 per cent in epidermoid cysts (*n* = 1) recurred.

In those with malignant tumours, all-cause 5-year mortality at the time of the study was 28 per cent (seven patients). Of those patients, six were symptomatic at presentation (85.7 per cent) and all of them developed metastatic or recurrent disease. Symptomatic presentation was not associated with long-term mortality (*P* = 0.262). The histological diagnosis in these patients was as follows: malignant change in tailgut cysts, adenocarcinoma in duplication cyst, chordoma in two patients, endometrial carcinoma, leiomyosarcoma, and liposarcoma.

## Discussion

The main findings of this study are that most patients affected by this tumour are female and present symptomatically with pain or symptoms related to a pelvic mass. Most tumours are located below S3 and are amenable to a transperineal approach for surgical management. Preoperative MRI provides good sensitivity and specificity for the detection of urovascular or pelvic sidewall involvement. Major complications after surgery are rare, the most common histological finding is a tailgut cyst, and solid tumours pose a higher risk of malignancy. R1 resection and symptomatic presentation are not associated with recurrence or mortality respectively.

The management of retrorectal tumours are lacking in national and international guidelines. There is continued debate as to whether surgical intervention is always required and whether biopsies are a safe and useful clinical tool in this situation. Perhaps as MRI techniques improve, this will enhance surgical planning before surgery, help to determine which surgical techniques are best in these diverse group of tumours, and how to safely manage patients after surgery.

In this patient cohort, preoperative MRI was found to be highly specific regarding urovascular and pelvic sidewall involvement of the tumour when assessed intraoperatively by the operating surgeon. This is important as some recommendations have suggested that lesions that invade into adjacent structures require *en bloc* resections, whereas lesions that abut surrounding structures may undergo a trial of dissection for circumferential resection. Our study enforces the use of preoperative MRI imaging. This study details experience from a centre specialized in managing retrorectal tumours; thus, wider application of this requires an established practice for reporting urovascular or pelvic sidewall involvement.

Histological subtypes of tumours after surgery were statistically more likely to be malignant if they were solid in nature, compared with cystic tumours; however, nearly 10 per cent of cystic tumours were still found to be malignant in nature. Hopper *et al.* suggested that cystic lesions should be conservatively managed with surveillance imaging^12^ and that solid lesions should be percutaneously biopsied where possible. In this patient cohort, biopsy was found to produce an incorrect or incomplete diagnosis in nearly one-third of patients despite current literature supporting its use^8,15^. In this series, surgical management of these tumours is safe with good outcomes when managed in specialized centres by surgical teams with relevant expertise.

The introduction of questionnaires in outpatient departments to assess functional outcomes and chronic pain may be beneficial in identifying this and providing a holistic approach to the management of retrorectal tumours.

This study has limitations with its retrospective assessment of patients in a single centre but still represents a large cohort of a relatively rare and heterogeneous disease. Intraoperative interpretation of the imaging findings (MRI/CT) is highly subjective and as such correlation between preoperative MRI findings and specimen histology should be considered a limitation of the study.

PRRTs can be safely excised with minimal complications. This study brings into question the conservative management of cystic tumours and given the risk of solid tumour malignancy, supports surgical management. MRI was highly specific in predicting urovascular and pelvic sidewall involvement but interpretation must be aligned with local expertise as an accurate method of preoperative planning. The surgical management of PRRTs delivers good oncological outcomes with limited co-morbidity and mortality.

## Data Availability

The data underlying this article are available upon request from the corresponding author.
